# Programmatic Mapping to Estimate Size, Distribution, and Dynamics of Key Populations in Kosovo

**DOI:** 10.2196/11194

**Published:** 2019-03-05

**Authors:** Dafina Gexha Bunjaku, Edona Deva, Luljeta Gashi, Pranvera Kaçaniku-Gunga, Carly A Comins, Faran Emmanuel

**Affiliations:** 1 National Institute of Public Health Department of Epidemiolo Pristina, Kosovo Albania; 2 Community Development Fund Kosovo Albania; 3 Bloomberg School of Public Health Department of Epidemiology Johns Hopkins University Baltimore, MD United States; 4 Centre for Global Public Health University of Manitoba Winnipeg, MB Canada

**Keywords:** HIV, program development, Kosovo, prevention and control, vulnerable populations, geographic mapping, epidemiology

## Abstract

**Background:**

The burden of an HIV epidemic in Kosovo lies among the key populations (KPs) of female sex workers (FSWs), men who have sex with men (MSM), and people who inject drugs (PWIDs). The available interventions for KPs are fragmented and lack sufficient and appropriate granularity of information needed to develop large-scale outreach programs.

**Objective:**

The aim of this study was to estimate the size and distribution of these populations to create evidence for developing action plans for HIV prevention.

**Methods:**

The programmatic mapping approach was used to collect systematic information from key informants, including geographic and virtual locations in 26 municipalities of Kosovo between February to April 2016. In level 1, information was gathered about KPs’ numbers and locations through 1537 key informant interviews within each municipality. Level 2 involved validating these spots by conducting another 976 interviews with KPs congregating at those spots. Population size estimates were calculated for each spot, and finally a national-level estimate was developed, which was corrected for duplication and overlaps.

**Results:**

Of the estimated 6814 MSM (range: 6445 to 7117), nearly 4940 operate through the internet owing to the large stigma and discrimination against same-sex relationships. Geo-based MSM (who operate through physical spots) congregate at a few spots with large spot sizes (13.3 MSM/spot). Three-fourths of the MSM are distributed in 5 major municipalities. Fridays and Saturdays are the peak days of operation; however, the number only increases by 5%. A significant number are involved in sex work, that is, provide sex to other men for money. PWIDs are largely geo-based; 4973 (range: 3932 to 6015) PWIDs of the total number of 5819 (range: 4777 to 6860) visit geographical spots, with an average spot size of 7.1. In smaller municipalities, they mostly inject in residential locations. The numbers stay stable during the entire week, and there are no peak days. Of the 5037 (range: 4213 to 5860) FSWs, 20% use cell phones, whereas 10% use websites to connect with clients. The number increases by 25% on weekends, especially in larger municipalities where sex work is mostly concentrated. Other than a few street-based spots, most spots are establishments run by pimps, which is reflective of the highly institutionalized, structured, and organized FSW network.

**Conclusions:**

This study provides valuable information about the population size estimates as well as dynamics of each KP, which is the key to developing effective HIV prevention strategies. The information should be utilized to develop microplans and effectively provide HIV prevention services to various KPs.

## Introduction

### Background

Kosovo is the smallest country in the Balkans region of Europe and, in terms of registered HIV cases, has the smallest HIV epidemic in the World Health Organization (WHO) Regional Office for Europe (WHO/Europe) [1,2]. A total of 97 HIV infections in Kosovo have been registered since the first reported case in 1986, and 41 AIDS-related deaths have been reported until 2015 [3]. The possibility of a high proportion of undiagnosed infections makes it difficult to estimate the overall HIV prevalence accurately and to confirm whether HIV incidence has remained stable. As no estimation process for people living with HIV has been carried out to date, based on current testing figures, it is difficult to make an estimate with any degree of accuracy [4].

Despite the small epidemic size, it is likely that the highest burden of HIV lies among various key populations (KPs), especially in men who have sex with men (MSM) and people who inject drugs (PWIDs), as seen in many neighboring countries [5]. The previous integrated biological and behavioral surveys (IBBS) rounds showed low numbers of HIV cases; however, one needs to consider that data were collected at only a couple of sites, and the study recruited a very small sample size mostly through convenience sampling [6,7]. HIV rates were low, but Hepatitis C rates among PWIDs remains a cause for concern, as it is a predictor of how widespread the HIV could become among PWIDs [8].

To date, the HIV epidemic in Kosovo has remained insignificant, yet there is a strong potential for its growth, particularly among MSM and PWIDs [9]. The experience working with these populations has demonstrated that effective interventions built around a population focus not only protect and engage members of these communities but also make a major contribution to averting a wider epidemic [10,11]. Substantial progress has been made to meet the strategic objectives and subobjectives of Kosovo HIV/AIDS strategy for 2009 to 2014 [12]. However, to improve the response to the new HIV/AIDS strategic targets for the period 2017 to 2021 [13], future interventions need to focus on KPs and should be based on WHO recommendations concerning KPs [14]. So far, the available interventions for KPs are fragmented, lack sufficient and appropriate coverage, and need a much more scientific base for program development. From a program planning perspective, it is essential to first quantify the size of KPs, understand their subtypes, and identify locations where they can be found [15].

### Objectives

On the basis of these principles, this study aimed to develop size estimates of KPs in Kosovo and provide the HIV prevention program with grassroot-level information to improve program design and coverage. The key objectives were to quantify the size of KPs, understand their subtypes, and identify locations where they can be found. Data from this study generated evidence for developing action plans for HIV prevention interventions tailored to KPs’ needs to scale up the HIV response.

## Methods

### Programmatic Mapping Overview

In epidemiological terms, the mapping methodology employed within this study resembled a cross-sectional survey to identify KPs in the epidemiological context of person, place, and time. The study was conducted in 26 of the 38 municipalities in Kosovo. The remaining municipalities were excluded because of security issues or political unrest. Data were collected from February to April 2016. During the preparation phase, KP communities and networks were engaged to discuss and develop procedures that ensured no harm was caused to the KP. This engagement with KPs was critical and consistent, with the approach of *nothing for us without us* through an assessment of the risks and benefits of programmatic mapping. Other important elements of the preparation phase included recruitment of field teams, training of the teams comprehensively in a 4-day training workshop on all aspects of field work, and pretesting and finalization of data collection tools. Other than the core training, the field teams were also provided a 2-day refresher training in between the 2 phases of field data collection.

### Geo-Mapping Approach

The broad methodological approach included 2 sequential phases. Level 1 (L1) was a systemic process of gathering information from secondary key informants on geo-locations where KPs congregate to meet sexual partners or inject drugs. Secondary key informants are people who are not KPs themselves but are knowledgeable of the presence and operations of KPs, for example, bar tenders, cab drivers, pimps, and massage parlor workers etc. A total of 1537 interviews were conducted in L1. The entire study area was divided geographically into smaller data collection units referred to as *zones*. The basic geo-administrative divisions in Kosovo called municipalities were considered as zones for this study. Data were collected in 26 out of 38 municipalities in Kosovo, as municipalities having political unrest and/or security issues were excluded. The municipalities of Prishtina and Prizren were further divided into 5 and 3 zones subsequently to a total number of 32 study zones where data were collected. The number of interviews within each municipality was kept between 35 and 60, based on population density and the geographic span of each zone. The primary outcome of L1 was the development of a spot list for each KP type within each zone. The following phase, level 2 (L2), included validation of spots identified in L1 for KP presence and activity as well as an in-depth profiling of each spot. L2 involved conducting interviews with KP informants, that is, FSWs, MSM, and PWIDs operating at the spots listed during L1 mapping. All respondents interviewed were older than 18 years and provided informed consent. Level 2 is considered to be the *validation stage*, where sites/spots mentioned by KIs in L1 are validated, and new spots are identified. Most spots for MSM and PWIDs were validated, whereas for FSW spots, the validation process was conducted only in spots where access was possible and a social mobilizer was available to facilitate the validation process. Residential spots were also not validated owing to issues of confidentiality. Members of the sex worker community were employed as *social mobilizers* who facilitated access and entry at various sex work spots. Social mobilizers connected with network operators or venue managers (eg, brothel, bars, and night clubs etc) and, after their consent, met the KP members operating at those venues to conduct a short interview. For data collection, teams of at least two (an interviewer and a social mobilizer) visited the identified hotspot and randomly selected a member of the KP present at the spot to conduct the interview. The social mobilizer facilitated the process by identifying and encouraging the KP members to participate in the study. The interviewer then formally conducted the interview after taking informed consent. The information was noted on an L2 form, which collected information on the type of spot, number of KP members who operate at the spot on a usual or peak day and obtained more specific information on the timings and days of operation. It also inquired the number of spots each KP member utilized to adjust for duplications. A total number of 976 spots were validated in L2. This included 255 spots for FSWs, 226 spots for MSM, and 495 spots for PWIDs.

### Web Mapping Approach for Men Who Have Sex With Men

Geographic mapping captures only the visible segment of KPs who operate at publicly accessible venues. Consultations with the MSM community revealed that it was important to account for the less visible/frequently active MSM who meet their clients/partners exclusively by other means (eg, through internet websites and mobile phone–based geo-social networking apps (GNA) [16,17]. Geographic mapping of MSM spots was therefore supplemented with virtual mapping of MSM who find same-sex partners primarily through these virtual sites.

The process involved working closely with the MSM community and making a list of all such MSM dating websites and mobile phone–based GNAs as the first step. These sites included global sites, county-specific sites, as well as Facebook and WhatsApp groups specific to the country. A total of 2 virtual mappers (VMs) who were knowledgeable about the use of such websites and apps were hired from the MSM community. A user profile was created at all active sites to be able to browse the websites/apps regularly. VMs logged in at each active internet site/GNA site 3 times a day for a period of 2 weeks to gather information about the total number of registered users, the number online at the particular time of visit, and the number of new registrations each day. To further understand these Web operations, the VMs contacted various virtual users randomly, introducing the research and encouraging them to participate in the study. Those who agreed to participate were invited for a face-to-face interview at a time and place of their convenience. During the interview, information was gathered on all websites/apps the MSM are registered with, multiple registrations with different identity documents at each site, the number of contacts made at each website, as well as use of geo-locations to look at overlap between virtual and geo-spaces. Although a high nonresponse was noticed (nearly one-thirds of the MSM contacted refused to participate), a sample of 84 MSM who operate through these sites were interviewed.

### Data Management and Analysis

All data collected in the field were entered in a database developed in Microsoft Excel. The team leaders, along with the data manger, were responsible for all aspects of data quality and consistency. All spots were provided a unique code, and data were checked for consistency (eg, missing information, spelling mistakes, and outliers etc) by the field supervisors before forms were submitted to the data management team. Data entry was done at the project office in Pristina, under the supervision of a database supervisor.

KP size estimates were calculated for each spot. As a first step, all reported minimum and maximum KP estimates were summed up to get a range of estimate for each spot, which were rolled up into municipality estimates, while adjusting for duplication, that is, multiple spots visited by the same KP and also for invisible KPs who do not come to spots, using the following formula, where *E*_*i*_=the adjusted estimated number of the KP, si=crude estimate of KPs for the spot; *p*_*i*_=proportion of population who visit more than 1 spot in a day, and *m*_*i*_=average number of spots visited in a day.

Ei= si(1 – pi) +

1

The municipality estimates were aggregated into national-level estimates after adjusting for invisibility factor. Finally, average spot sizes (number of KPs per spot) were determined as well as the density of each KP per 1000 adult men or women was also calculated. The adult male and female population of Kosovo was used as a denominator [18], whereas the numerator was the estimate produced by this study.

### Ethical Considerations

This appraisal was designed to meet international ethical protocols by taking effective measures to avoid risk, protect individuals’ rights, and ensure safety of all study participants. Ethical approval for the study was taken from the Ethical Review Board at the Ministry of Health, Kosovo. In addition, specific measures were taken to ensure the safety of the field teams and KPs. Data were collected only in municipalities which were deemed safe and received support from the local police. The KP community was given the power to make decisions on how this project was implemented, and all their concerns and suggestions were duly incorporated in the research protocol. Recruitment of participants was conducted only after describing the study procedures and obtaining informed consent. A nonidentifying coding system was used to track study data while assuring nondisclosure of participants’ identities. All participants were provided information on existing services and were linked to the health and social services that are available for this community.

## Results

### Estimated Numbers of Key Populations

A total number of 17,670 KP members were identified in Kosovo. The largest KP found within the country was MSM, followed by PWIDs and FSWs. Aside from a difference in size estimation, stark differences were found surrounding the operational dynamics of each population, the type and location of spots frequented, and the alternative methods for seeking partners and/or engaging in risk activity.

This study estimated a total of 5037 (range: 4213 to 5860) FSWs in Kosovo (Table 1). Although an estimated number of 4163 out of 5037 (82.6%) FSWs operate through geographic spots, nearly one-fifth (1058) are clandestine and remain unseen. These FSWs do not come to geo-physical locations but use other forms of contact to connect with their clients, that is, cell phones, internet hookups, or through personal contacts with pimps etc. Approximately, a tenth of the FSWs use the internet to find clients, but a significant overlap with geo-spots and cell phone hookups was reported. PWIDs were estimated at 5819 (range: 4777 to 6860). A larger number, that is, 4974 of 5819 were geo-spot–based, spread over 847 spots. Although this study identified no females in PWIDs, they were probably a part of the unseen PWIDs who operate undercover, not frequenting geographic locations. MSM were found to be the largest KP in Kosovo, with a total estimate of 6814 (range: 6445 to 7117). A smaller number of MSM operated at geo-spots (1874 MSMs congregate at 141 geographical spots), whereas a much larger number of MSM (ie, 4940 out of 6814) reported to operate through internet sites and mobile apps. Interviews conducted at L2 confirmed that Facebook is the most popular networking site, whereas Grinder and Planet Romeo are the most used mobile apps. A significant proportion of MSM provide sexual services to other men in return for money and can thus be regarded as male sex workers (MSWs). An estimated number of 731 (range: 595 to 865) MSWs were found to be distributed throughout the country, mostly operating through geo-spots. Results of the analysis which calculated KP density (number of FSWs per 1000 adult females and number of MSM per 1000 adult males, etc) showed approximately 9 FSWs per 1000 adult females, 10 PWIDs per 1000 adult men, and 12 MSM per 1000 adult men in Kosovo.

### Municipality Distribution of Key Populations in Kosovo

**Figure 1:**
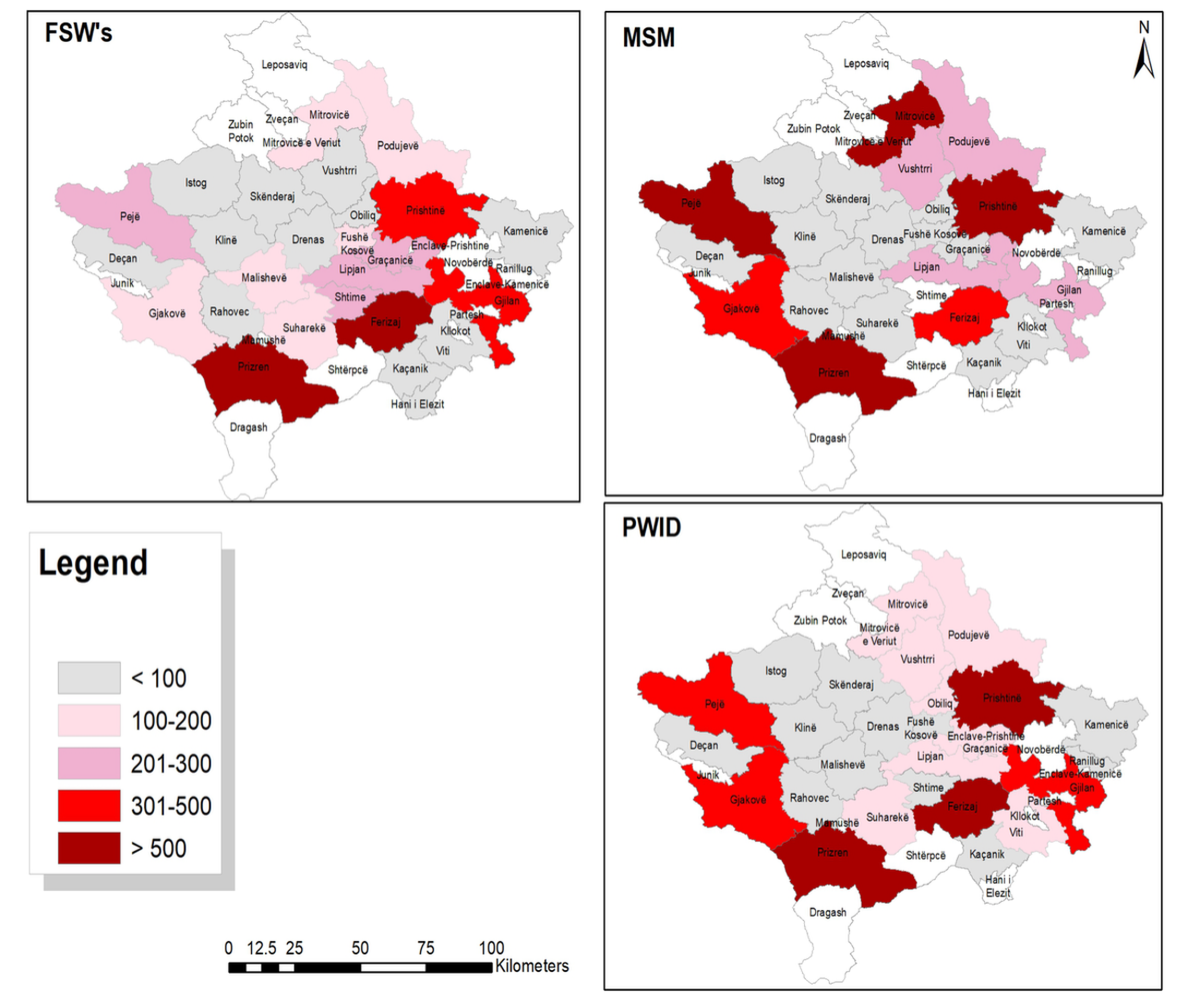
Distribution of female sex workers (FSWs), men who have sex with men (MSM), and people who inject drugs (PWIDs) by municipalities in Kosovo in 2016.

Figure 1 shows the distribution of FSWs, PWIDs, and MSM in various municipalities of Kosovo. Ferizaj, Prizren, Prishtinë, and Gjilan were municipalities with the highest number of FSWs having 16% (806/5037), 13.1 % (660/5037), 9.9% (499/5037), and 8.8% (443/5037) of the estimated FSWs, respectively. More than half of the municipalities in Kosovo had an insignificant number of FSWs (less than 2% of the total FSWs). The distribution of PWIDs varied significantly by municipality. Nearly half of the PWIDs concentrate in 3 municipalities of Prishtinë, Ferizaj, and Prizren accounting for 24.5%, 15.2%, and 9.6%, respectively. MSM seem to concentrate in fewer municipalities with Prishtinë and Prizren, reporting the highest number of MSM, that is, 2613 and 1277 MSM, respectively. In Prishtinë, of the 2613 MSM identified, 84.8% (2215/2613) MSM were Web-based, whereas in Prizren, 58.2% (743/1276) were Web-based. Other municipalities with higher numbers of MSM included Mitrovicë, Gjakovë, and Pejë.

**Table 1:** Estimated number of female sex workers (FSWs), people who inject drugs (PWIDs), and men who have sex with men (MSM) in Kosovo in 2016.

Characteristics	FSWs^a^	PWIDs^b^	MSM^c^
Estimated number^d^	5037 (4213-5860)	5819 (4777-6860)	6814 (6445-7117)
Estimated number at geo-spots	4163 (3482-4843)	4974 (3932-6015)	1874 (1570-2177)
Estimated number of nongeo-spot/Web-based	1058	845	4940
Estimated number of male sex workers	—^e^	—	731 (595-865)
Number of geo-spots	790	847	141
Adult population	566560^f^	568903^g^	568903^g^
Number of KPs^h^ per 1000 adults	8.9 per adult females	10.2 per adult males	12.0 per adult males

^a^FWSs: female sex workers.

^b^PWIDs: people who inject drugs.

^c^MSM: men who have sex with men.

^d^The sum of geo-spot–based and nongeo-spot–based estimates might not add up to the total estimated number because of adjustments made in the final estimate to account for duplication.

^e^Not applicable.

^f^Adult female population.

^g^Adult male population.

^h^KPs: key populations.

### Spot Typology and Operational Dynamics

Table 2 shows the spot typologies and operational dynamics of FSWs, PWIDs, and MSM hotspots of Kosovo. FSWs mostly operate through geo-spots having an average spot size of 5.3 FSWs per spot, with significant differences across municipalities. The peak days of activity for FSWs was reported to be Friday and Saturday, Nearly three fourth FSWs operate in evenings, whereas half of them operate at night as well. More than two-thirds of FSWs find clients at restaurants with or without music and coffee shops, where they work as hostesses. The spots for PWIDs were different from FSW spots. These included abandoned buildings, establishments, public transport stops or parks, streets, and some residential buildings as well. Street spot was the largest spot typology followed by abandoned buildings, which accounted for 41.1% (2044/4974) `and 31.5% (1567/4974) of PWIDs, respectively. A much smaller proportion of MSM is visible on physical spots. Over half of MSM spots comprised street spots/parks and bus stop spots etc. Although the average spot size of MSM is large (13.3 MSM per spot), great variation existed across municipalities and by spot type. The average spot size for restaurant/coffee shop spots was high, with an estimated 19.3 MSM per spot, whereas the spot size for street spots was 11.3. Although peak days for MSM was reported to be on weekends, that is, Fridays and Saturdays, all days of the week reported substantial MSM activity.

**Table 2:** Spot typologies and operational dynamics for various key populations in Kosovo in 2016.

Mapping		FSWs^a^		PWIDs^b^		MSMs^c^	
		Number of spots	Estimates	Number of spots	Estimates	Number of spots	Estimates
Estimated number		790	4163	847	4974	141	1874
Spot size		5.3	5.3	5.9	5.9	13.3	13.3
**Type of spots**							
	Abandoned buildings	0	0	240	1567	0	0
	Hotel/motel/guest house	38	238	155	804	3	22
	Public transport stop	13	62	42	261	63	709
	Residential place	4	16	63	298	11	95
	Restaurant with live music	225	1326	0	0	0	0
	Restaurant/coffee shop	330	1584	0	0	18	348
	Salons/casino/shops	66	273	0	0	20	318
	Streets/open spaces/park	114	664	347	2044	26	382
Peak day of operation		Friday, Saturday		All week		Friday, Saturday	
**Peak time of operation, (n/N)**							
	Morning (before 12 noon)	11.4 (29/481)		71.0 (431/607)		12.1 (16/132)	
	Afternoon (12-5 PM)	41.2 (105/481)		87.2 (529/607)		44.7 (59/132)	
	Evening (5-9 PM)	51.8 (132/481)		57.8 (351/607)		59.8 (79/132)	
	Night (9 PM onward)	78.0 (199/481)		20.9 (127/607)		39.4 (52/132)	

^a^FWSs: female sex workers.

^b^PWIDs: people who inject drugs.

^c^MSM: men who have sex with men.

## Discussion

### Summary

Although there have been some efforts in Kosovo in the past to estimate KP size and understand their operational dynamics [6,7,19], this study has significantly contributed to enhance our knowledge. Not only were we able to estimate the size of various KPs in Kosovo, we also identified geo-locations where HIV transmission may be maximum and developed a pragmatic typology of KPs and spots, which improved our understanding of these populations. There is enough evidence provided to understand the distribution and structure of KPs as well as know their size and operational dynamics, which is central to develop a prevention response to halt the progression of HIV and AIDS [11,15].

Our study followed a simple community-led approach, ensuring active leadership and involvement of the KPs themselves in validating estimates. A total number of 6814 MSM, 5819 PWIDs, and 5037 FSWs were identified in Kosovo. Other than FSW estimates, the estimated numbers for both MSM and PWIDs are much lower than what have been calculated through previous research [6,7,19]. The previous estimates were derived from IBBS data using multiplier technique methods. IBBS samples were collected from Prishtina and Prizren alone, which were extrapolated to develop national estimates for MSM and PWIDs. Our study found that the number of KPs in large urban centers, that is, Pristina, Ferizaj, and Prizren, was many folds in comparison with smaller municipalities, and thus extrapolating rates from urban centers to smaller municipalities could have led to an overestimation of the size of PWIDs and MSM in Kosovo. In contrast to previous research, the estimates derived from this study followed a comprehensive exercise based on data collected at the grassroot level, led by KPs. While calculating these estimates, we adjusted for KPs visiting multiple spots and also for hidden KPs who do not come to spots. Finally, we triangulated the results with program data from service delivery programs and verified it with nongovernmental organizations (NGOs) working with these populations. The triangulation process included carefully looking at monitoring data from the programs and conducting discussion with peer workers and program managers to validate various key spots and number of KPs in various municipalities.

This research revealed in-depth information on the structure, subculture, and operational dynamics of each KP and found striking differences between them. This study identified 7 different types of geographic spots where FSWs in Kosovo congregate, find sexual partners, or engage in sexual activities. FSWs in Kosovo are centered around restaurants/coffee shops with live music and hotels/motels etc with a number of typologies involved; each having its own operational dynamics and prevention needs. Nearly all FSWs are a part of a wider but hidden network that functions undercover and are largely managed by network operators and pimps. Although sex workers are visible at a few spots, the network that operates them is extremely organized and well-connected and mostly keeps the sex work clandestine. This nature of sex work in Kosovo is reflective of the illegality of sex work and high prevalence of sex trafficking into and through the country [20].

PWIDs represent a small proportion of an overall large population of drug users in Kosovo [19]. Unlike FSWs, PWIDs have a much wider distribution across most municipalities surveyed. Injecting drugs typically occurs in abandoned places/houses, open spaces, streets, parks etc and mostly happen during times when these places are not frequented by people. Abandoned buildings were also found to be locations where PWIDs mostly purchase drugs and inject. A predominate percentage of PWIDs visit geo-spots after noon and continue injecting through the entire day. As injecting is an everyday phenomenon, there were no peak days reported, and the estimated number of PWIDs did not change. Most injecting occurs at homes, and street-based drug injecting is not very common; thus, PWIDs are also covert and not visible. Major cities such as Pristina, Ferizaj, etc were identified as more open societies, whereas Peja, Podujeva, Skenderaj, and Gjakova were identified as very stigmatized, and PWIDs are not prepared to publicly identify themselves. Nonetheless, because of the overall drug use patterns and availability of injectable drugs in Kosovo [19], PWIDs make up a large KP identified in this mapping activity. MSM have distinct features, which makes them notably different from PWIDs and FSWs. Owing to the large stigma and discrimination faced generally, MSM concentrate in a fewer municipalities, that is, Prishtinë, Prizren, Mitrovicë, among others, which are considered urban and more open-minded. The higher number of MSM in these areas are representative of the higher number of students and MSM-friendly establishments such as night clubs. Most MSM operate discreetly through internet websites or mobile phones and are not visible to the general community in most municipalities, as is seen in many other countries globally [16,21]. After connecting with other peer members, they would go to either public spots or discreet locations, for example, homes, abandoned buildings, etc and usually meet after the sunset and during the late hours of the night. During discussions, MSM emphasized that they do not feel safe during the night hours, especially in the small cities, as they might be identified easier. Among MSM who do visit geographic spots, a large majority do not meet within their own municipality/city but travel to neighboring cities for physical contact.

Overall, this study has provided valuable information about the operational typologies and dynamics of these populations, which is the key to developing effective HIV prevention strategies. As part of utilization of the results, the knowledge gained from this study could be used to strategize target regions and towns where provision of services would be most effective and cost beneficial [22]. Of the key strengths of this approach, 1 lies not only in its development of estimates but also in providing a consequential distribution of KP members at different spots. Thus, larger spots with a high number of KP sizes should be the focus of prevention programs and could be the hubs of service delivery [23]. It is difficult to fully comprehend the extent and organizational dimensions of sex work or same sex without a long engagement and trust-building period with KPs. With such numbers of KPs reported, there is a need to continue a focused HIV prevention program for these populations.

### Limitations

There were few limitations the study encountered regarding the geographic-spot validation process. Security issues and prohibited entry surrounding PWIDs and FSW spots inhibited the validation of various spots. Moreover, misclassification of exposures may also have occurred leading to an over or under representation of the study population. This could have resulted in an over classification of drug users as PWIDs or under classification of MSM who only engage in sexual activity with men occasionally or experimentally; thus, various drug users who share the same spots as PWIDs could have been included in the estimated PWIDs numbers, and MSM who do not engage regularly in MSM activities might have been missed in the overall estimates of MSM.

### Conclusions

Overall, this study has shown that a substantial number of KPs are present in Kosovo, comprising of MSM, PWIDs, and FSWs. Each KP has a unique geographic distribution and operational dynamics by which they seek partners and/or engage in risk activity. Despite all limitations, this study’s findings can guide program planners to develop appropriate HIV program implementation strategies and enhance coverage. Knowledge gained can be used to develop macroplans to strategically target regions/town and microplans to strategically deliver services, as well as to develop and strengthen the structural components of the HIV prevention programs within Kosovo.

## Abbreviations

FSW: female sex worker
GNA: geo-social networking apps
IBBS: integrated biological and behavioral survey
KP: key population
L1: Level 1
L2: Level 2
MSW: male sex worker
MSM: men who have sex with men
NGO: nongovernmental organization
PWIDs: people who inject drugs
VM: virtual mapper
WHO: World Health Organization
